# Parental mosaicism in another case of Dravet syndrome caused by a novel *SCN1A* deletion: a case report

**DOI:** 10.1186/s13256-016-0854-2

**Published:** 2016-03-29

**Authors:** Rajech Sharkia, Holger Hengel, Ludger Schöls, Muhammad Athamna, Peter Bauer, Muhammad Mahajnah

**Affiliations:** 1 The Triangle Regional Research and Development Center, P. O. Box-2167, Kfar Qari’, 30075 Israel; 2 Beit-Berl Academic College, Beit-Berl, 44905 Israel; 3 German Research Center for Neurodegenerative Diseases (DZNE), 72076 Tübingen, Germany; 4 Department of Neurodegenerative Diseases and Hertie-Institute for Clinical Brain Research, University of Tübingen, Hoppe-Seyler Str. 3, 72076 Tübingen, Germany; 5 Institute of Medical Genetics and Applied Genomics, University of Tübingen, 72076 Tübingen, Germany; 6 Child Neurology and Development Center, Hillel-Yaffe Medical Center, 38100 Hadera, Israel; 7 Bruce and Ruth Rappaprt Faculty of Medicine, Technion, 31096 Haifa Israel

**Keywords:** Dravet syndrome, Genetic counseling, Paternal mosaicism, *SCN1A* gene

## Abstract

**Background:**

Dravet syndrome, a rare genetic disorder with early-onset epileptic encephalopathy, was first described by Dravet in 1978. Dravet syndrome is most frequently caused by various mutations of the *SCN1A* gene encoding the type 1 subunit of the neuronal voltage-gated sodium channel.

**Case presentation:**

Two sisters of a non-consanguineous Palestinian family from the Arab community in Israel attended our child development and pediatric neurology clinic due to recurrent seizures and developmental delay. Genomic DNA was extracted from peripheral blood lymphocytes of all family members and a *SCN1A* mutation in exon 10 was revealed by Sanger sequencing in both affected siblings but not in the parents. Our data present a case of Dravet syndrome caused by a novel heterozygous *SCN1A* deletion (c.1458_1465delCTCTAAGT) in two affected siblings. Our findings add to the spectrum of mutations known in the *SCN1A* gene and confirm parental mosaicism as a mechanism relevant for transmission of this disease.

**Conclusions:**

These cases confirm parental mosaicism in the transmission of Dravet syndrome and add to the spectrum of known mutations of the *SCN1A* gene. Repeated reports on parental mosaicism should remind us that there is a risk of recurrence even if the mutation is apparently *de novo*.

## Background

Dravet syndrome (DS), a rare genetic disorder with early-onset epileptic encephalopathy (EIEE), was first described by Dravet in 1978 [1]. It is also known in the literature as “severe myoclonic epilepsy of infancy.” Initially, the electroencephalogram (EEG) is generally normal, but shows characteristically generalized spike-wave activity later in the disease [2]. A febrile seizure is usually the initial manifestation. Afterwards, different seizure types evolve, such as tonic-clonic and myoclonic seizures, as well as absences. DS is associated with ataxia, slowed psychomotor development, intellectual disability, and is often refractory to anticonvulsant medication [3, 4].

DS is an autosomal dominant disease that is mainly caused by various mutations of the *SCN1A* gene encoding the type 1 subunit of the neuronal voltage-gated sodium channel [5]. Mutations reported to cause DS are usually truncating as well as missense mutations in *SCN1A* resulting in haploinsufficiency. Most of the *SCN1A* mutations are *de novo* mutations; this explains why parents and siblings are usually not affected. However besides *de novo* cases, there have been reports of parental mosaicism in the literature [6–10].

In this study, we report a novel *SCN1A* deletion in DS transmitted by parental mosaicism. The novel heterozygous deletion (c.1458_1465delCTCTAAGT) was shown by direct sequencing of the *SCN1A* gene in two affected siblings. The clinical phenotype revealed typical features of DS in both patients, with minor differences. The reoccurrence of the same mutation in the two children with no detection of the mutation in parents’ lymphocytes indicates a genetic mosaicism in one of the parents.

## Case presentation

**Fig. 1:**
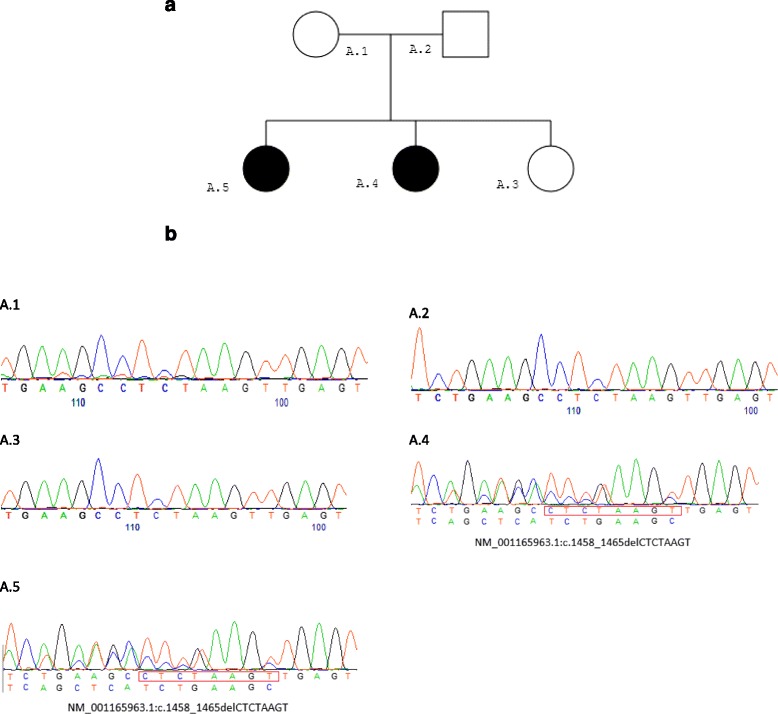
**a** Family pedigree: the parents are not related. *Squares* indicate males and *circles* females. Affected patients are filled *black*. **b** Electropherogram showing the same heterozygous deletion in *SCN1A* in both affected children (A4 and A5), but no deletion in the parents or the healthy child (A1, A2, A3). Sequences have been reverse complemented to show the forwarding sequence. The deleted sequence (delCTCTAAGT) is given in a *red box* in the two patients A4 and A5

This research was prospectively reviewed and approved by the ethics committee in our center. Two siblings of a non-consanguineous Palestinian family (Fig. 1a) from the Arab community in Israel attended our child development and pediatric neurology clinic due to recurrent seizures and developmental delay.

### Patient 1

The first patient is a 7-year-old Palestinian girl from the Arab community in Israel; she was born to non-consanguineous parents after a full-term pregnancy by normal delivery with a normal birth weight of 3.1 kg. According to her parents she had normal development until the age of 1 year. At this age she was hospitalized because of a generalized tonic-clonic convulsion during febrile disease. She was discharged with the diagnosis of simple febrile convulsions. A few months later she had recurrent episodes of febrile convulsions. She underwent an EEG recording, which was normal. At the age of 2 years she had several attacks of non-febrile generalized tonic-clonic seizures; every attack lasted for 3 to 7 minutes followed by 10 to 15 minutes of a postictal state. Another interictal EEG after sleep deprivation and photic stimulation was normal. Furthermore, karyotype testing and brain magnetic resonance imaging (MRI) were carried out when she was 2-years old and found to be normal. She also underwent cerebrospinal fluid examination for protein, glucose, lactate levels and cell count, yielding normal results. Routine metabolic screening was normal. Ophthalmologic and hearing examinations were normal. She was further followed by our pediatric neurology and child development clinic. Her examination revealed no dysmorphic features and no visceral enlargement. She showed mild motor and cognitive delay but could stand and walk with support, and could speak up to 15 words with good communication abilities. Her neurologic examination revealed normal tone and normal tendon reflexes of her lower and upper limbs. Babinski sign was normal. Her cranial nerves, sensory perception and cerebellar function were normal. For the seizures she was treated with valproic acid and topiramate, with good response. She attended special education kindergarten, with speech therapy and occupational therapy. At the most recent examination, at the age of 7 years, she had normal intelligence and attended a regular school with assistance because of learning disabilities and attention deficit disorder.

### Patient 2

The second patient is a 3.5-year-old Palestinian girl from the Arab community in Israel who is the younger sister of patient 1. She was born after a full-term pregnancy by normal delivery, with normal birth weight of 3.0 kg. At the age of 1 month, a small ventricular septal defect was diagnosed, but it resolved spontaneously at 1 year of age. At the age of four months she was hospitalized for elective umbilical hernia surgery. Immediately after the surgery she had fever due to a urinary tract infection leading to a focal convulsion involving her right upper and lower extremities. The convulsion lasted 5 minutes. She underwent medical investigation including EEG recording and brain MRI, which were normal. Later she had recurrent right focal seizures with fever. At the age of 1.5 years she also had recurrent non-febrile focal seizures of her right extremities, generalized tonic-clonic seizures and focal seizures with secondary generalization. She was treated with carbamazepine, valproic acid, rectal diazepam and levetiracetam, with partial improvement. At this time, because of the recurrent non-febrile generalized tonic-clonic seizures, she underwent several EEG recordings after sleep deprivation and photic stimulation, which were normal. Furthermore, karyotype testing and brain MRI were carried out, and found to be normal. Laboratory tests, including cerebrospinal fluid examination for protein, glucose, lactate levels and cell count, yielded normal results. Routine metabolic screening was normal. Ophthalmologic and hearing examinations were normal.

She was followed by our pediatric neurology and child development clinic. Her examination at the age of 3.5 years revealed no dysmorphic features and no visceral enlargement. She showed a severe cognitive delay; her gross motor abilities were intact but she had severe speech and language deficits. Her neurologic examination revealed normal tone and normal tendon reflexes of her lower and upper limbs. Babinski sign was normal. Her cranial nerves, sensory perception and cerebellar function were normal. Her developmental evaluation revealed mild to moderate intellectual disability and she is attending special education.

### Molecular genetic analysis

Genomic DNA was extracted from peripheral blood lymphocytes of all family members, using standard procedures. A *SCN1A* mutation in exon 10 was revealed by Sanger sequencing in the index patient. The truncating mutation (NM_001165963.1:c.1458_1465delCTCTAAGT; NP_001159435.1:p.Ser487GlufsTer6) is novel to the best of our knowledge (Fig. 1b). We then sequenced the entire exon 10 of all family members using Sanger sequencing. To our surprise, both affected children had the same mutation, whereas the mutation was not detected by Sanger sequencing in either of the parents. To exclude the possibility of a different biological father, a paternity test was performed using microsatellite analysis, and it confirmed paternity (data not shown).

## Discussion

DS is most frequently caused by various mutations of the *SCN1A* gene encoding the subunit 1 of the neuronal voltage-gated sodium channel [5]. To date, about 600 *SCN1A* mutations have been identified in DS [11]. The majority of *SCN1A* mutations are *de novo*, and most of them are of paternal origin [12].

Our finding of a novel truncating mutation (NM_001165963.1:c.1458_1465delCTCTAAGT; NP_001159435.1:p.Ser487GlufsTer6) adds to the spectrum of *SCN1A* mutations causing DS. The pathomechanism in known mutations is assumed to be a loss of function of the mutated allele; this matches with the truncating type of mutation found in our family and provides additional evidence of the pathogenicity of the 8-base pairs (bp) deletion. Moreover, our results demonstrate that the mechanism of transmission in this family is most likely a parental mosaicism. As both children but neither of the parents carry the mutation it is likely that one of the parents carries a germline mutation that is not detectable in lymphocytes due to mosaicism. Unfortunately, no semen of the father and no other tissues were available for further analyses of the mosaic pattern. There might even be a low mutation load in one of the parents that could not be detected due to limitations of the sensitivity of Sanger sequencing. The reoccurrence of the same mutation in the two children with no detection of the mutation in the parents’ lymphocytes indicates that even apparent *de novo* mutations can reoccur in siblings, most likely because of gonadal mosaic mutations. We recommend genetic counseling and pre-implantation genetic diagnosis (PGD) when parents have a child with a *SCN1A* mutation, since there is an increased chance of bearing another baby with the same mutation. It is important for genetic counselors to know that the mechanism of genetic mosaicism with somatic or germline mutations seems to be more common than expected [6].

Both patients showed similar clinical manifestations including epilepsy and cognitive disabilities. Despite these similarities, some differences exist. Patient 1 has well-controlled epilepsy and normal intelligence, but with attention deficit and learning disabilities. Patient 2 has a mild to moderate intellectual disability and attends a special education school. She has intractable recurrent non-febrile generalized tonic-clonic seizures. These cases illustrate that the same mutation in the same family can cause variable phenotypes of DS.

## Conclusions

These cases confirm parental mosaicism in the transmission of DS and add to the spectrum of known *SCN1A* mutations. Repeated reports on parental mosaicism should remind us that there is a risk of recurrence even if the mutation is apparently *de novo*.

## Consent

Written informed consent was obtained from the parents of our patients for publication of this case report and the accompanying images. A copy of the written consent is available for review by the Editor-in-Chief of this journal.

## Abbreviations

bp: Base pairs
DS: Dravet syndrome
EEG: Electroencephalogram
EIEE: Early-onset epileptic encephalopathy
MRI: Magnetic resonance imaging
PGD: Pre-implantation genetic diagnosis.
